# Expensive today but cheaper tomorrow: lifetime costs of an active middle ear implant compared to alternative treatment options

**DOI:** 10.1007/s10198-024-01743-6

**Published:** 2024-12-06

**Authors:** Markus Krohn, Klaas Kiesewetter, Annika Buchholz, Bettina Schlick, Susan Busch, Thomas Lenarz, Anke Lesinski-Schiedat, Hannes Maier, Cornelia Batsoulis, Michael Urban, Steffen Flessa

**Affiliations:** 1 Chair of General Business Administration and Health Care Management, University of Greifswald, Greifswald, Germany; 2 MED-EL Medical Electronics, Innsbruck, Austria; 3 Department of Otolaryngology, Hannover Medical School, Hannover, Germany; 4 Clinic for Laryngology, Rhinology and Otology, Hannover Medical School, Hannover, Germany; 5 MED-EL Medical Electronics, MED-EL Research Center, Hannover, Germany

**Keywords:** Middle ear implant, Cost analysis, Lifetime cost, Monte-Carlo-Simulation, Hearing-improvement surgeries, I10, I11, I13, C15

## Abstract

**Background:**

When choosing between different treatment options, implants often appear too costly. However, this perspective does not take future costs into account. This article evaluates lifetime costs for different surgical interventions to treat hearing loss.

**Methods:**

The analysis focused on three groups from the perspective of health insurers. Group 1 comprises patients who have only been implanted with a middle ear implant. Patients in Group 2 had already undergone middle ear surgery to improve hearing prior to the implantation of a middle ear implant. Group 3 consists of patients who were treated exclusively with hearing-improvement surgeries (no implant). The lifetime costs were calculated using the Monte Carlo simulation. The inputs were based on medical data from a maximum-care hospital and data from the German healthcare system.

**Results:**

Based on an average observation period of 26.73 years, the lifetime costs amounted to 28,325€ for group 1, 32,187€ for group 2 and 28,381€ for group 3. While the mean values between groups 1 and 3 appear comparable, group 1 has a significantly lower standard deviation (G1 vs. G3: 6120€ vs. 10,327€).

**Discussion/conclusion:**

Choosing a treatment option can be a complex medical decision and impose a substantial economic burden for the statutory health insurance. Hence, treatment decisions should be patient-centred at first but also including a shared-decision making on economic feasibility, whether proposed treatment alternatives are likely to be successful and economically reasonable.

## Introduction

Several treatment options are usually considered as part of any medical decision-making process. For example, treatment options for progressive hearing loss may include hearing-improvement surgeries (tympanoplasties) or middle ear implants. While typical surgery for hearing loss costs around 3700 € (example DRG D30A) [1], the costs for a middle ear implant are higher at around 13,000 € [2]. At first, the implant appears to be disadvantageous from an economic perspective. However, there are other possible effects that may arise in the further course of treatment; therefore, costs should be calculated over the entire lifespan based on medical data.

According to the World Report on Hearing by the World Health Organization (WHO), hearing loss is the fourth highest cause of disability globally, with an estimated annual cost of more than 980 billion dollars [3]. In Germany, the annual cost of health disorders caused by hearing impairments were estimated to be € 2.65 billion in 2011 [4]. Current data suggest that approximately 5% of the world’s population or 430 million people require auditory rehabilitation to address their disabling hearing loss. It is estimated that by 2050 more than 700 million people – or 1 in every 10 people – will suffer a disabling hearing loss (hearing loss greater than 35 decibels (dB) in the better-hearing ear). According to the meta study “Hearing Loss – Numbers and Costs” published by hear-it AISBL, there are approximately 34.4 million people in the European Union living with a disabling hearing loss, with 22.6 million people remaining untreated. This results in an annual overall cost of 185 billion Euros or 8200 Euros per affected person [5]. Furthermore, approximately 10 million people in the EU suffer a hearing loss of 50 dB or greater, with around 5.5 million of these still untreated.

In 2021 in Germany alone, approximately 4.5 million people (5.35%) had a disabling hearing loss, according to the most recent data from the Institute for Health Metrics and Evaluation (IHME) [6]. In 2019, Löhler et al. investigated six studies involving hearing disorders in Germany, showing a broad range of hearing loss prevalence (between 16 and 25%) with no standardized definition of hearing loss [7]. Also in 2019, Schmucker et al. conducted a systematic review to complement the results of Löhler et al. reporting a prevalence of hearing loss in the child and adolescent German population ranging from 0.1 to 128 per 1000 children and adolescents [8]. Self-reported hearing loss at a higher threshold (> 30 dB HL) ranged between 2.4 and 5.2% [8]. Another epidemiological study on hearing status in northwest Germany (HÖRSTAT) estimated the prevalence of hearing loss, as defined by the World Health Organization, to be 16%, which is in line with the number of participants who had a hearing level greater than 40 dB in the “hear-it AISBL” article [9]. It is obvious that disabling hearing loss negatively affects multiple aspects of an individual’s life when left unaddressed or when the individual’s communication needs are unsupported.

For many years, standard treatment for conductive and/or mixed hearing loss has consisted of middle ear surgeries with or without the replacement of defective ossicles (tympanoplasty) and if possible, the application of a hearing aid. Middle ear surgeries, such as a tympanoplasties and/or mastoidectomies (surgeries to the mastoid bone for infection and the tympanic membrane), are frequently performed with excellent results for the treatment of infectious middle ear diseases with or without implanting passive ossicular prostheses to restore hearing. Middle ear surgeries are primarily concerned with resolving the discharging pathology, as in the case of patients with chronic otitis media (COM), or with the complete eradication in case of cholesteatomas. As the pathologies have a high chance of reoccurrence, these procedures may usually require repeated surgeries. Success of middle ear surgeries can be linked to the functional restoration of the tympanic membrane, with high recurrence rates between 50 and 100%, including a variety of different and frequently interrelated causes of failures, making surgical prognosis very difficult, often resulting in a severe mixed hearing impairment in patients with COM often not able to wear their hearing-aids. In addition, hearing aid usage in such patients may lead to radical cavity infections or recurrent myringitis if sealed too tightly [10]. Even when patients undergo multiple appropriate middle ear surgeries, hearing improvement sometimes remains inadequate.

These patients with unsuccessful conventional hearing rehabilitation and a variety of middle ear conditions can be treated effectively using an electromagnetically active middle ear implant such as the Vibrant Soundbridge (VSB), as the anatomical conditions in such cases often impede an adequate acoustic coupling. Active middle ear implants (AMEIs) are medical devices intended to treat hearing loss by stimulation of the middle and inner ear structures. This study investigated the use of the Vibrant Soundbridge (VSB) (MED-EL Austria), the world’s, up to now, most utilized AMEI. With more than 25 years of experience in restoring hearing for thousands of people around the world, the VSB is suitable for different types of hearing loss and is also approved for children. It is implanted in cases of sensorineural hearing loss when the fitting of conventional hearing aids is not possible because of chronic otitis externa, as well as in cases of conductive or combined hearing impairment when conventional tympanoplasties and passive ossiculoplasties cannot sufficiently improve hearing.

Although surgeries to improve hearing and active middle ear implants are both highly relevant in this patient population and both may be used for auditory rehabilitation in the same patient. There exist very few studies that compare the lifetime costs of these treatment options. Such studies are necessary for policymakers and surgeons to inform themselves about the cost efficacy of different interventions. In this paper we intend to fill this research gap.

## Methodology

The analysis compared the costs of three care alternatives from the perspective of health insurers. In the following model, there are specific durations between the interventions. These are derived from the medical data. Within these time periods, the treatment success, e.g. hearing improvement, is defined as comparable between the options. The analysis does not include the results of hearing measurements (e.g. in dB) or health-related quality of life. This assumption is essential for a meaningful comparison of lifetime costs:

The following three groups of patients were defined:

1. Patients who received a middle ear implant directly, without prior hearing-improvement surgery.
2. Patients who underwent one or more hearing-improvement surgeries before receiving a middle ear implant.
3. Patients who had more than one hearing-improvement surgeries but no middle ear implant.

The overall lifetime costs will be investigated considering several input parameters. A Monte Carlo simulation will be used as the methodological tool. In addition to the average cost per intervention, statements can be made about the benefit of the intervention based on the uncertainty of the individual input values. In particular, the parameters “implantation age/remaining lifetime” and the influence of the number of interventions will be looked at.

### Treatment data

The treatment data in this retrospective study originated from the hospital information system of a maximum care hospital. Only the data of patients that gave their consent during treatment was analysed. Groups 1 and 2 consisted of all patients implanted with a VSB at the Hannover Medical School, Hannover, Germany, between 1997 and 2021, selected from the internal database for middle ear implants. Other types of middle ear implants were not included in this study. This resulted in 389 patients. Patients were excluded if their last appointment was earlier than 2019, as well as for having multimorbidity’s or private insurance.

Subjects were selected and grouped based on: AMEI without prior corrective hearing-improvement surgery (group 1: n = 100) or corrective hearing-improvement surgery before AMEI implantation (group 2: n = 51).

The patients were then screened so that each patient had an individual incident list with specific dates of their inpatient and outpatient visits related to their AMEI. The inpatient stays included appointments such as implantation, revision, or re-implantation. For group 2, the previous hearing-improvement operations were added. Outpatient visits included regular annual check-ups and processor fittings, visits for complications and/or hearing problems. These specific inpatient and outpatient events were then registered in SAP to confirm that the appointment in the internal database had indeed taken place and to specify which OPS and DRG codes of these appointments were used for billing purposes.

For group 3, an SAP query was generated at the central department of MHH IT Services Applications, Enterprise Documents and Content, MHH Information Technology for patients with the OPS codes 5–195.90, .91, .92, .93 and 5–197.2. This determination of relevant patients in group 3 is done according to OPS codes (German procedure classification “Operations- und Prozedurenschlüssel - OPS”). In this step, group 2 serves as the basis for the selection of procedures. Based on the data in group 2, it was determined which hearing-improving surgeries are to be considered common before a VSB implant. The five OPS codes mentioned resulted from this analysis. Patients who received at least one of the procedures mentioned and were surgically treated between 2018 and 2021 and had statutory health insurance were included. Exclusion criteria were bilateral care and multimorbidity, since in these cases the data of the individual visit in SAP (especially for outpatient visits) could no longer be comprehensibly assigned to one specific pathology. This resulted in 211 patients, out of which 100 were randomly selected. Like the patients in groups 1 and 2, their visits were also registered in SAP so that an incident list with corresponding ICD codes, OPS codes and DRG codes could be created for each patient.

### Cost data

Relevant cost data of the individual services were taken from the German health care system. For inpatient treatments, the hearing-improvement surgery costs were taken from the observed set of per-case flat rates (aG-DRG), including additional nursing revenues from the per-case flat rate catalogue [11]. For non-rated aG-DRGs, revenue values were used that were individually negotiated between health insurers and hospitals [2, 12–19]. These values also include the costs of the implant. For outpatient stays, the university’s outpatient flat rate of the hospital the data originated from was used. Consequently, all cost data are real data and not assumptions or estimates.

### Simulation

The exported medical data was then validated, and the group classification was checked, as well as the age distribution between the groups. From the medical data, distribution identification was used to determine the theoretic distributions of the input parameters for individual model input parameters [20]. Normal distribution, truncated normal distribution, log-normal, exponential and uniform distribution were all tested. The patient’s age at first intervention, duration between hearing-improvement surgeries, duration between processor upgrades, and duration of care with hearing-improvement surgeries before VSB implantation were all considered. Furthermore, based on the observed probabilities for revision or re-implantation (depending on the duration of use) in VSB patients, a triangular distribution was calculated.

We then performed a Monte Carlo simulation with the findings of the data preparation. The Monte Carlo simulation includes treatment data and cost data. Based on the individual input values and the identified distributions, the costs for all three patient groups were determined. For each group, 10,000 simulation runs were performed. The result of the simulation is thus not only a fixed individual result, but rather a probability distribution of the target values [21]. The aim is to ensure that the simulation model represents reality in the best possible way. The quality of the model is checked by comparing the model results with the treatment data from the hospital of origin.

The individual steps of the simulation are explained below. The calculation is described in text form. The description covers the key aspects of the calculation. The factors described in text form are highlighted in bold in Tables 1, 2, 3. The formal implementation is shown in Tables 1, 2, 3. In addition to the aspects described, these tables contain all the calculation steps, including all the necessary intermediate steps and auxiliary calculations. Table 4 shows the input parameters.

*Group 1: Patients who were directly fitted with a middle ear implant without first undergoing a hearing-improvement surgical intervention.*

Based on the distribution of age at initial intervention (“AFI”) and the life expectancy (“LEX”), which is defined as fixed, the treatment period (“TOP” = total observation period) was calculated. For this period, the costs of the first intervention (in this case, implantation of VSB) (“G1_CFI (group 1 - cost first intervention)”), the costs for re-implantations (“G1_CRI (group 1 - cost re-implantation)”), the costs for revisions (“G1_CRE (group 1 - cost revision)”), the costs for audio processor upgrades (“G1_APU (group 1 - audio processor upgrade)”) and the costs for an annual check-up were included for each simulation run. These add up to the lifetime cost of group 1 (“G1_LTC”).

The costs for the first intervention include the costs of implantation (“ISB (Implantation Vibrant Soundbridge)”) based on the determined distribution and three outpatient follow-up appointments (“FOA (flat-rate outpatient appointments)”).

The costs for re-implantations were similarly composed. The costs for “ISB” and “FOA” were also used here. The only difference is that the number of “FOA” appointments was reduced to two, since the third appointment was covered by the annual check-up appointment in the model. Furthermore, the time of a first or second re-implantation results from the lifetimes of the implants (“LTS (Lifetime Vibrant Soundbridge) or LTS1, LTS2 and LTS3”). The model was limited to a maximum of two re-implantations. Possible resulting model errors due to a possible increased number of required re-implantations was indicated by (“EP_LTS_G1 (error probability lifetime Vibrant Soundbridge group 1)”). This value thus partially describes the model quality. Furthermore, a discount rate (“DIR”) of 5% was applied for all costs arising in the future. This corresponds to the Hanoverian consensus and is taken into account in all parts of the model [22]. The number of implantations results from the initial implantation plus the number of necessary re-implantations and is defined as "NOI_G1 (number of implantations group 1)”.

The costs for revisions include the costs of an inpatient revision stay (“REV”) according to the determined distribution plus an additional “FOA” appointment. The interval between necessary revisions is derived from the determined distribution. These distributions are defined as “TTR (time to revision) or TTR1, TTR2, TTR3, TTR4”. The model allows for a maximum of three revisions. The model error, which describes the need for additional revisions, is defined as “EP_TTR_G1 (error probability time to revision group 1)”. The number of revisions is defined as “NOR_G1 (number of revisions group 1)”.

The cost of the audio processor upgrades was calculated from the cost of one audio processor (“APU”), taking into account the period between updates (“TAP”) and the total duration of care (“TOP”). The minimum time to upgrade is six years, as billing to health insurance is mainly possible after six years [23]. The time between the completed sixth year of use and the change to a new processor was derived from the observed data. This follows an exponential distribution. The number of processor upgrades is defined as “NOP_G1” (number of processor upgrades).

The total cost of annual inspections (FOA) was calculated as the present value over the “TOP” period. Furthermore, the outputs “DR_LTS” and “DR_TTR” were generated. These describe the default rates of the implants (LTS) after 1, 10 and 25 years of use and the revision probability (TTR) after 1, 5 and 10 years. The values were used to determine the model quality. Here, the data from the hospital information system are compared with the results of the simulation runs. The outputs including all correction steps are shown in Table 1. The outputs explicitly presented in the method description are printed in bold.

**Table 1:** Simulation model – group 1

Output	Equation
TOPuB1	if(round(LEX-AFIa) > (LEX-MIA),round(LEX-AFIb),round(LEX-AFIa))
TOPuB2	if(TOPuB1 > 77;round(LEX-AFIc);TOPuB1)
TOPlB1	if(TOPuB2 < 1;round(LEX-AFIb);TOPuB2)
TOPlB2	if(TOPlB1 < 1;round(LEX-AFIc);TOPlB1)
TOPlB3	if(TOPlB2 < 1;1;TOPlB2)
TOPuB3	if(TOPlB3 > (LEX-MIA);(LEX-MIA);TOPlB3)
**TOP**	TOPuB3
**AFI**	LEX-TOP
LTS1lB	if(LTS1 < 0;0;LTS1)
LTS2lB	if(LTS2 < 0;0;LTS2)
LTS3lB	if(LTS3 < 0;0;LTS3)
**EP_LTS_G1**	TOP-LTS1lB-LTS2lB-LTS3lB [upper spec limit = 0]
**DR_LTS1**	round(LTS1lB-0,5) [lower spec limit = 1]
**DR_LTS10**	round(LTS1lB-0,5) [lower spec limit = 10]
**DR_LTS25**	round(LTS1lB-0,5) lower spec limit = 25]
**NOI_G1**	1 + if(LTS1lB > = TOP;0;1) + if((LTS1lB + LTS2lB) > = TOP;0;1)
TTR1lB	if(TTR1 < 0;0;TTR1)
TTR2lB	if(TTR2 < 0;0;TTR2)
TTR3lB	if(TTR3 < 0;0;TTR3)
TTR4lB	if(TTR4 < 0;0;TTR4)
TTR1uB	TTR1lB
TTR2uB	TTR2lB
TTR3uB	TTR3lB
TTR4uB	TTR4lB
**EP_TTR_G1**	TOP-TTR1uB-TTR2uB-TTR3uB-TTR4uB [upper spec limit = 0]
**DR_TTR1**	round(TTR1uB-0.5) [lower spec limit = 1]
**DR_TTR5**	round(TTR1uB-0.5) [lower spec limit = 5]
**DR_TTR10**	round(TTR1uB-0.5) [lower spec limit = 10]
**NOR_G1**	if(TTR1uB > = TOP;0;1) + if((TTR1uB + TTR2uB) > = TOP;0;1) + if((TTR1uB + TTR2uB + TTR3uB) > = TOP;0;1)
TTU	TTUa + TTUb
**TAP**	round(TTU)
**NOP_G1**	if(TOP > = TAP;1;0) + if(TOP > = (2*TAP);1;0) + if(TOP > = (3*TAP);1;0) + if(TOP > = (4*TAP);1;0) + if(TOP > = (5*TAP);1;0) + if(TOP > = (6*TAP);1;0) + if(TOP > = (7*TAP);1;0) + if(TOP > = (8*TAP);1;0) + if(TOP > = (9*TAP);1;0) + if(TOP > = (10*TAP);1;0) + if(TOP > = (11*TAP);1;0) + if(TOP > = (12*TAP);1;0)
**G1_CFI**	(ISB + 3*FOA)/((1 + DIR)^0)
**G1_CRI**	if(LTS1lB < TOP;1;0)*((ISB + 2*FOA)/((1 + DIR)^(round(LTS1lB-0.5)))) + if((LTS1lB + LTS2lB) < TOP;1;0)*((ISB + 2*FOA)/((1 + DIR)^((round(LTS1lB + LTS2lB-0.5)))))
**G1_CRE**	if(TTR1uB < TOP;1;0)*((REV + FOA)/((1 + DIR)^(round(TTR1uB-0.5)))) + if((TTR1uB + TTR2uB) < TOP;1;0)*((REV + FOA)/((1 + DIR)^((round(TTR1uB + TTR2uB-0.5))))) + if((TTR1uB + TTR2uB + TTR3uB) < TOP;1;0)*((REV + FOA)/((1 + DIR)^((round(TTR1uB + TTR2uB + TTR3uB-0.5)))))
**G1_APU**	if(TOP > = TAP;1;0)*(APU/((1 + DIR)^TAP)) + if(TOP > = (2*TAP);1;0)*(APU/((1 + DIR)^(2*TAP))) + if(TOP > = (3*TAP);1;0)*(APU/((1 + DIR)^(3*TAP))) + if(TOP > = (4*TAP);1;0)*(APU/((1 + DIR)^(4*TAP))) + if(TOP > = (5*TAP);1;0)*(APU/((1 + DIR)^(5*TAP))) + if(TOP > = (6*TAP);1;0)*(APU/((1 + DIR)^(6*TAP))) + if(TOP > = (7*TAP);1;0)*(APU/((1 + DIR)^(7*TAP))) + if(TOP > = (8*TAP);1;0)*(APU/((1 + DIR)^(8*TAP))) + if(TOP > = (9*TAP);1;0)*(APU/((1 + DIR)^(9*TAP))) + if(TOP > = (10*TAP);1;0)*(APU/((1 + DIR)^(10*TAP))) + if(TOP > = (11*TAP);1;0)*(APU/((1 + DIR)^(11*TAP))) + if(TOP > = (12*TAP);1;0)*(APU/((1 + DIR)^(12*TAP)))
**G1_TSC**	FOA*((((1 + DIR)^(TOP-1))-1)/(((1 + DIR)^(TOP-1))*DIR))
**G1_LTC**	G1_CFI + G1_CRI + G1_CRE + G1_APU + G1_TSC

*Group 2: Patients who received a middle ear implant after one or more hearing-improvement middle ear surgeries.*

Group 2 patients underwent hearing-improvement surgeries prior to VSB implantation. Consequently, the first intervention consisted of a hearing-improvement surgery (“G2_CFI (group 2 - cost first intervention)”). Possible further hearing-improvement surgeries (“G2_CFS (group 2 - cost further surgery) as well as the subsequent treatment with VSB have to be taken into account. This includes the costs of the first implantation (“G2_VSB (group 2 - cost implantation VSB)”), the costs for re-implantations (“G2_CRI (group 2 - cost re-implantation)”), the costs for revisions (“G2_CRE (group 2 - cost revision)”) and the costs for audio processor upgrades (“G2_APU (group 2 – audio processor upgrade)”). In addition, there are costs for an annual service appointment (“G2_TSC”) for the entire time. Together this results in the lifetime costs of group 2 (“G2_LTC”). During the period of treatment with hearing-improving surgeries, costs for possible hearing aids are not included. This point is addressed in the discussion.

The content of G2_LTC is identical to G1_LTC. Furthermore, the contents of G2_VSB are also identical to G1_CRI, G2_CRI to G1_CRI, G2_APU to G1_APU and G2_CRE to G1_CRE. There are only two changes to be considered. The time of use of the VSB needs to be shortened because hearing-improvement operations were performed before the VSB. Furthermore, the number of FOA appointments is reduced to two for G2_VSB because the third appointment is included by G2_LTC. The previous observation time (“TOP”) is now split into “THI (time of hearing-improving interventions)” and “TSB (time of Vibrant Soundbridge)”. THI results from the determined distribution with a maximum value of 25 years. This maximum definition is necessary because group 2 patients change to a VSB. If no maximum value were defined, treatment sequences could arise that correspond to group 3. The number of operations results from the determined success duration between hearing-improvement operations and THI. The duration of success of surgery was defined as “DSH1, DSH2, … (duration success hearing-improving surgery)”. The number of hearing-improvement surgeries was limited to six including initial intervention and is defined as NHI_G2. The probability of needing more surgeries is described as EP_NHI_G2 (error probability number of hearing-improvement surgeries group 2). The cost of hearing-improvement surgery was taken from the distribution of the observed aG-DRG set. Furthermore, two FOA appointments were included after each surgery.

The outputs and equations including all correction steps are shown in Table 2. The outputs explicitly presented in the method description are printed in bold.

**Table 2:** Simulation model (additions) – group 2

Output	Equation
THIlB	if(round(THIt) < TOP,round(THIt),TOP)
**THI**	if(THIlB < 25,THIlB,25)
**TSB**	TOP-THI
**NHI_G2**	1 + if(DSH1 < THI,1,0) + if((DSH1 + DSH2) < THI,1,0) + if((DSH1 + DSH2 + DSH3) < THI,1,0) + if((DSH1 + DSH2 + DSH3 + DSH4) < THI,1,0) + if((DSH1 + DSH2 + DSH3 + DSH4 + DSH5) < THI,1,0)
**EP_NHI_G2**	THI-DSH1-DSH2-DSH3-DSH4-DSH5-DSH6
**G2_CFI**	(HIS1 + 2*FOA)/((1 + DIR)^0)
**G2_CFS**	if(DSH1 < THI,1,0)*((HIS2 + FOA)/((1 + DIR)^round(DSH1-0,5))) + if((round(DSH1 + DSH2)) < THI,1,0)*((HIS3 + FOA)/((1 + DIR)^round(DSH1 + DSH2-0,5))) + if((round(DSH1 + DSH2 + DSH3)) < THI,1,0)*((HIS4 + FOA)/((1 + DIR)^round(DSH1 + DSH2 + DSH3-0,5))) + if((round(DSH1 + DSH2 + DSH3 + DSH4)) < THI,1,0)*((HIS5 + FOA)/((1 + DIR)^round(DSH1 + DSH2 + DSH3 + DSH4-0,5))) + if((round(DSH1 + DSH2 + DSH3 + DSH4 + DSH5)) < THI,1,0)*((HIS6 + FOA)/((1 + DIR)^round(DSH1 + DSH2 + DSH3 + DSH4 + DSH5-0,5)))
**G2_VSB**	(ISB + 2*FOA)/((1 + DIR)^THI)
**G2_CRI**	if(LTS1lB < TSB;1;0)*((ISB + 2*FOA)/((1 + DIR)^round(THI + LTS1lB-0,5))) + if((LTS1lB + LTS2lB) < TSB;1;0)*((ISB + 2*FOA)/((1 + DIR)^round(THI + LTS1lB + LTS2lB-0,5)))
NOI_G2	1 + if(LTS1lB > = TSB,0,1) + if((LTS1lB + LTS2lB) > = TSB,0,1)
**G2_CRE**	if(TTR1uB < TSB,1,0)*((REV + FOA)/((1 + DIR)^round(THI + TTR1uB-0,5))) + if((TTR1uB + TTR2uB) < TSB,1,0)*((REV + FOA)/((1 + DIR)^round(THI + TTR1uB + TTR2uB-0,5))) + if((TTR1uB + TTR2uB + TTR3uB) < TSB,1,0)*((REV + FOA)/((1 + DIR)^round(THI + TTR1uB + TTR2uB + TTR3uB-0,5)))
NOR_G2	if(TTR1uB > = TSB,0,1) + if((TTR1uB + TTR2uB) > = TSB,0,1) + if((TTR1uB + TTR2uB + TTR3uB) > = TSB,0,1)
**G2_TSC**	G1_TSC
**G2_APU**	if(TSB > = TAP;1;0)*(APU/((1 + DIR)^(TAP + THI))) + if(TSB > = (2*TAP);1;0)*(APU/((1 + DIR)^(2*TAP + THI))) + if(TSB > = (3*TAP);1;0)*(APU/((1 + DIR)^(3*TAP + THI))) + if(TSB > = (4*TAP);1;0)*(APU/((1 + DIR)^(4*TAP + THI))) + if(TSB > = (5*TAP);1;0)*(APU/((1 + DIR)^(5*TAP + THI))) + if(TSB > = (6*TAP);1;0)*(APU/((1 + DIR)^(6*TAP + THI))) + if(TSB > = (7*TAP);1;0)*(APU/((1 + DIR)^(7*TAP + THI))) + if(TSB > = (8*TAP);1;0)*(APU/((1 + DIR)^(8*TAP + THI))) + if(TSB > = (9*TAP);1;0)*(APU/((1 + DIR)^(9*TAP + THI))) + if(TSB > = (10*TAP);1;0)*(APU/((1 + DIR)^(10*TAP + THI))) + if(TSB > = (11*TAP);1;0)*(APU/((1 + DIR)^(11*TAP + THI))) + if(TSB > = (12*TAP);1;0)*(APU/((1 + DIR)^(12*TAP + THI)))
NOP_G2	if(TSB > = TAP;1;0) + if(TSB > = (2*TAP);1;0) + if(TSB > = (3*TAP);1;0) + if(TSB > = (4*TAP);1;0) + if(TSB > = (5*TAP);1;0) + if(TSB > = (6*TAP);1;0) + if(TSB > = (7*TAP);1;0) + if(TSB > = (8*TAP);1;0) + if(TSB > = (9*TAP);1;0) + if(TSB > = (10*TAP);1;0) + if(TSB > = (11*TAP);1;0) + if(TSB > = (12*TAP);1;0)
**G2_LTC**	G2_CFI + G2_CFS + G2_VSB + G2_CRI + G2_CRE + G2_APU + G2_TSC
EP_LTS_G2	TSB-LTS1lB-LTS2lB-LTS3lB
EP_TTR_G2	TSB-TTR1uB-TTR2uB-TTR3uB-TTR4uB

*Group 3: Patients who received more than one hearing-improvement surgery but no middle ear implant.*

Patients in group 3 received hearing-improvement surgery throughout the observation period. The costs of this group consist of the costs of the first intervention (“G3_CFI”) plus the costs of further hearing-improvement surgeries (“G3_CFS”), as well as an assumed annual control visit whose total costs are represented by “G3_TSC”. G3_TSC corresponds to G1_TSC in terms of content. Together this results in the lifetime costs of group 3 (“G3_LTC”). The time between hearing-improvement operations corresponds to the value “DSH1, DSH2, …” already presented in group 2. The number of interventions, whose costs per intervention follow the same distribution as in group 2 (“HIS”), is limited in the model to 15 interventions (initial intervention plus a maximum of 14 follow-up interventions). The probability of higher need is described by EP_NHI_G3 (error probability number of hearing-improvement surgeries group 3). Consequently, group 3 corresponds to group 2 in terms of content, but the provision of hearing-improvement surgery takes place over the entire duration (“TOP”), not only during a short period (“THI”) before a subsequent VSB implantation. The outputs including all correction steps are shown in Table 3. The outputs explicitly presented in the method description are printed in bold. In addition, it should be noted for group 3 that no costs are included for the provision of hearing aids. This point is addressed in the discussion.

**Table 3:** Simulation model (additions) – group 3

Output	Equation
**G3_CFI**	(HIS1 + 2*FOA)/((1 + DIR)^0)
**G3_CFS**	if(DSH1 < TOP;1;0)*((HIS2 + FOA)/((1 + DIR)^round(DSH1-0.5))) + if((round(DSH1 + DSH2)) < TOP;1;0)*((HIS3 + FOA)/((1 + DIR)^round(DSH1 + DSH2-0.5))) + if((round(DSH1 + DSH2 + DSH3)) < TOP;1;0)*((HIS4 + FOA)/((1 + DIR)^round(DSH1 + DSH2 + DSH3-0.5))) + if((round(DSH1 + DSH2 + DSH3 + DSH4)) < TOP;1;0)*((HIS5 + FOA)/((1 + DIR)^round(DSH1 + DSH2 + DSH3 + DSH4-0.5))) + if((round(DSH1 + DSH2 + DSH3 + DSH4 + DSH5)) < TOP;1;0)*((HIS6 + FOA)/((1 + DIR)^round(DSH1 + DSH2 + DSH3 + DSH4 + DSH5-0.5))) + if((round(DSH1 + DSH2 + DSH3 + DSH4 + DSH5 + DSH6)) < TOP;1;0)*((HIS7 + FOA)/((1 + DIR)^round(DSH1 + DSH2 + DSH3 + DSH4 + DSH5 + DSH6-0.5))) + if((round(DSH1 + DSH2 + DSH3 + DSH4 + DSH5 + DSH6 + DSH7)) < TOP;1;0)*((HIS8 + FOA)/((1 + DIR)^round(DSH1 + DSH2 + DSH3 + DSH4 + DSH5 + DSH6 + DSH7-0.5))) + if((round(DSH1 + DSH2 + DSH3 + DSH4 + DSH5 + DSH6 + DSH7 + DSH8)) < TOP;1;0)*((HIS9 + FOA)/((1 + DIR)^round(DSH1 + DSH2 + DSH3 + DSH4 + DSH5 + DSH6 + DSH7 + DSH8-0.5))) + if((round(DSH1 + DSH2 + DSH3 + DSH4 + DSH5 + DSH6 + DSH7 + DSH8 + DSH9)) < TOP;1;0)*((HIS10 + FOA)/((1 + DIR)^round(DSH1 + DSH2 + DSH3 + DSH4 + DSH5 + DSH6 + DSH7 + DSH8 + DSH9-0.5))) + if((round(DSH1 + DSH2 + DSH3 + DSH4 + DSH5 + DSH6 + DSH7 + DSH8 + DSH9 + DSH10)) < TOP;1;0)*((HIS11 + FOA)/((1 + DIR)^round(DSH1 + DSH2 + DSH3 + DSH4 + DSH5 + DSH6 + DSH7 + DSH8 + DSH9 + DSH10-0.5))) + if((round(DSH1 + DSH2 + DSH3 + DSH4 + DSH5 + DSH6 + DSH7 + DSH8 + DSH9 + DSH10 + DSH11)) < TOP;1;0)*((HIS12 + FOA)/((1 + DIR)^round(DSH1 + DSH2 + DSH3 + DSH4 + DSH5 + DSH6 + DSH7 + DSH8 + DSH9 + DSH10 + DSH11-0.5))) + if((round(DSH1 + DSH2 + DSH3 + DSH4 + DSH5 + DSH6 + DSH7 + DSH8 + DSH9 + DSH10 + DSH11 + DSH12)) < TOP;1;0)*((HIS13 + FOA)/((1 + DIR)^round(DSH1 + DSH2 + DSH3 + DSH4 + DSH5 + DSH6 + DSH7 + DSH8 + DSH9 + DSH10 + DSH11 + DSH12-0.5))) + if((round(DSH1 + DSH2 + DSH3 + DSH4 + DSH5 + DSH6 + DSH7 + DSH8 + DSH9 + DSH10 + DSH11 + DSH12 + DSH13)) < TOP;1;0)*((HIS14 + FOA)/((1 + DIR)^round(DSH1 + DSH2 + DSH3 + DSH4 + DSH5 + DSH6 + DSH7 + DSH8 + DSH9 + DSH10 + DSH11 + DSH12 + DSH13-0.5))) + if((round(DSH1 + DSH2 + DSH3 + DSH4 + DSH5 + DSH6 + DSH7 + DSH8 + DSH9 + DSH10 + DSH11 + DSH12 + DSH13 + DSH14)) < TOP;1;0)*((HIS15 + FOA)/((1 + DIR)^round(DSH1 + DSH2 + DSH3 + DSH4 + DSH5 + DSH6 + DSH7 + DSH8 + DSH9 + DSH10 + DSH11 + DSH12 + DSH13 + DSH14-0.5)))
**G3_TSC**	G1_TSC
**EP_NHI_G3**	TOP-DSH1-DSH2-DSH3-DSH4-DSH5-DSH6-DSH7-DSH8-DSH9-DSH10-DSH11-DSH12-DSH13-DSH14-DSH15
**G3_LTC**	G3_CFI + G3_CFS + G3_TSC
NHI_G3	1 + if(DSH1 < TOP;1;0) + if((DSH1 + DSH2) < TOP;1;0) + if((DSH1 + DSH2 + DSH3) < TOP;1;0) + if((DSH1 + DSH2 + DSH3 + DSH4) < TOP;1;0) + if((DSH1 + DSH2 + DSH3 + DSH4 + DSH5) < TOP;1;0) + if((DSH1 + DSH2 + DSH3 + DSH4 + DSH5 + DSH6) < TOP;1;0) + if((DSH1 + DSH2 + DSH3 + DSH4 + DSH5 + DSH6 + DSH7) < TOP;1;0) + if((DSH1 + DSH2 + DSH3 + DSH4 + DSH5 + DSH6 + DSH7 + DSH8) < TOP;1;0) + if((DSH1 + DSH2 + DSH3 + DSH4 + DSH5 + DSH6 + DSH7 + DSH8 + DSH9) < TOP;1;0) + if((DSH1 + DSH2 + DSH3 + DSH4 + DSH5 + DSH6 + DSH7 + DSH8 + DSH9 + DSH10) < TOP;1;0) + if((DSH1 + DSH2 + DSH3 + DSH4 + DSH5 + DSH6 + DSH7 + DSH8 + DSH9 + DSH10 + DSH11) < TOP;1;0) + if((DSH1 + DSH2 + DSH3 + DSH4 + DSH5 + DSH6 + DSH7 + DSH8 + DSH9 + DSH10 + DSH11 + DSH12) < TOP;1;0) + if((DSH1 + DSH2 + DSH3 + DSH4 + DSH5 + DSH6 + DSH7 + DSH8 + DSH9 + DSH10 + DSH11 + DSH12 + DSH13) < TOP;1;0) + if((DSH1 + DSH2 + DSH3 + DSH4 + DSH5 + DSH6 + DSH7 + DSH8 + DSH9 + DSH10 + DSH11 + DSH12 + DSH13 + DSH14) < TOP;1;0)

### Model parameters

The following Table 4 shows the input parameters of the simulation model. The source of the data origin is described in the last column.

**Table 4:** Input values

Input name	Distribution	Parameters	Source
LEX – life expectancy	Fixed	Value: 81	[24] (mean value man and woman)
MIA – minimum implantation age	Fixed	Value: 5	[25]
AFIa, AFIb, AFIc – age at first intervention (for lower/upper bound correction)	Weibull	Shape: 244.32323 Scale: 2866.73542 Threshold: -2806.08613	own, based on medical data
FOA – flat-rate outpatient appointments	Fixed	Value: 145	own, based on MHH cost data
DIR – discount rate	Fixed	Value: 0.05	[22]
ISB – cost of implantation of Vibrant Soundbridge	Lognormal	Location: 9.57283 Scale: 0.07429	own, based on medical data and cost data mentioned before
LTS1, LTS2, LTS3 – lifetime Vibrant Soundbridge	Triangular	Lower: -3 Mode: 0 Upper: 400	own, based on medical data
TTR1, TTR2, TTR3 – time to revision	Triangular	Lower: -3 Mode: 0 Upper: 140	own, based on medical data
REV – cost revision	Lognormal	Location: 8.29047 Scale: 0.20121	own, based on medical data and cost data set
APU – cost audio processor	Fixed	Value: 5719.9	[26]
TTUa – time to upgrade APU minimum	Fixed	Value: 6	[23]
TTUb – additional time to upgrade APU	Exponential	Rate: 1.41988	own, based on medical data
THIt – Time of hearing-improving intervention total (for lower/upper bound correction)	Lognormal	Location: 0.70939 Scale: 1.3453	own, based on medical data
DSH1, DSH2, … DSH15 – duration of success of hearing-improving surgery	Weibull	Shape: 0.68361 Scale: 1.74601 Threshold: 0.28273	own, based on medical data
HIS1, HIS2, … HIS15 – cost of hearing-improving surgery	Lognormal	Location: 8.2339 Scale: 0.25662	own, based on medical data and cost data set

## Results

### Treatment duration (“TOP”)

In the model, the duration of care was calculated from the difference between a fixed life expectancy of 81 years and the age at first intervention according to distribution identification.

**Fig. 1:**
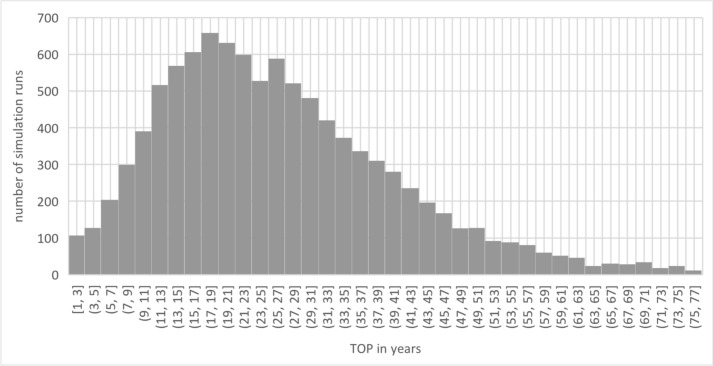
Total observation period (TOP) (source: own simulation)

This resulted in a “TOP” duration of 26.73 years, with a standard deviation of 13.94 years. This duration describes the period of lifetime costs. In group 1 it includes the care with VSB, and in group 3 the care by means of hearing-improvement operations. For group 2, this period was divided into the care by means of hearing-improvement operations (“THI”) and the treatment time with VSB (“TSB”). Figure 1 shows the distribution for “TOP”. In group 2, the duration of care with hearing-improvement surgery was 4.01 years (± 5.18), and for care with VSB 22.72 years (± 14.60). The sum of the durations again corresponds to TOP. Time in years is plotted on the x-axis, and the number of observations in the model is plotted on the y-axis.

### Model results of group 1

**Fig. 2:**
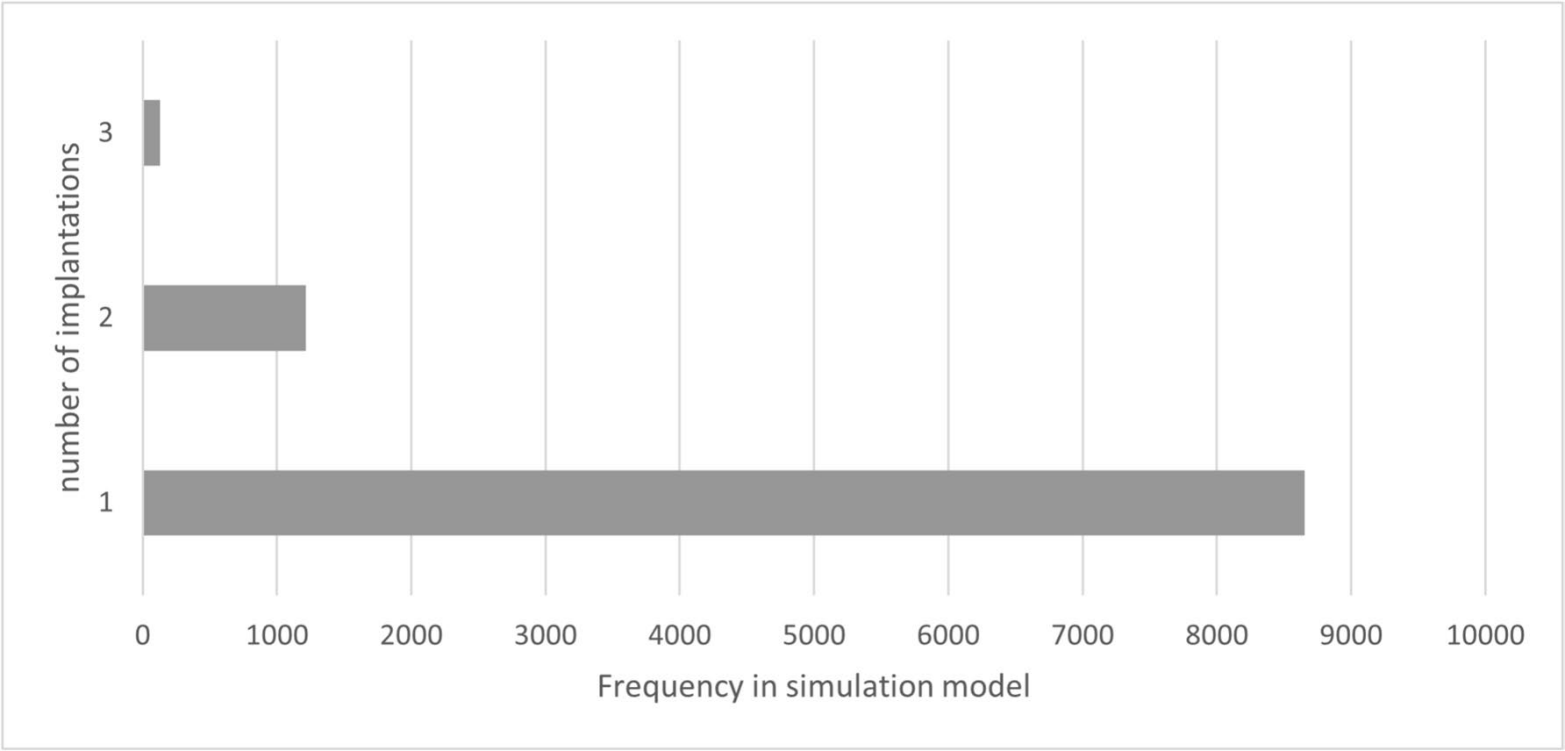
Number of implantations (including initial implantation) – group 1 (source: own simulation)

**Fig. 3:**
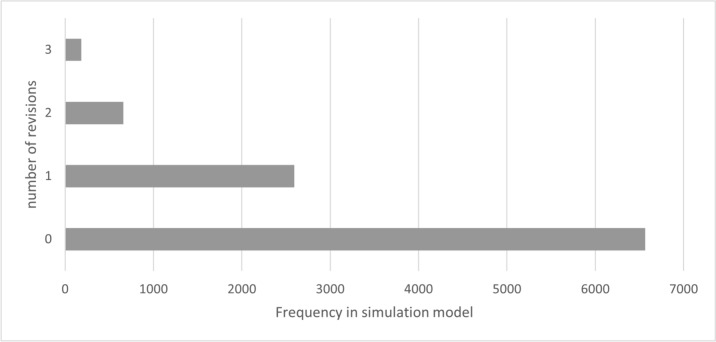
Number of revisions – group 1 (source: own simulation)

In group 1, care was provided exclusively with a VSB. During the 26.73 [± 13.94]-year treatment period, a mean of 1.15 [± 0.39] implantations were performed. The number of re-implantations over the entire lifetime is therefore 0.15, with 86.6% of patients requiring no re-implantation and 12.2% requiring only one re-implantation. The number of revision surgeries necessary over the entire period of care averaged 0.45 (± 0.70), with 65.6% of patients requiring no revision and 26.0% requiring only one revision. The average number of processor upgrades was 3.64 (± 2.19). The values shown relate to the overall model. Due to the distribution of “TOP”, 50% of patients in the model will have a VSB treatment duration of more than 25 years. The following figures show the number of implantations (Fig. 2) and revisions (Fig. 3).

The question arises as to whether the model can represent reality. To answer this, the comparison between model results and real data must be used. The model shows the following values for the failure probabilities of the implants (DR_LTS).

- Rate after one year: 1.12%
- Rate after ten years: 5.65%
- Rate after 25 years: 12.62%.

The following values were observed:

- Rate after one year: 1.00%
- Rate after ten years: 4.80%
- Rate after 25 years: 4.80% - No implant failures across all patients after the tenth year of use.

Thus, the model is close to reality in terms of failure rates, but more conservative than the real data. Higher treatment requirements are expected, a circumstance that can be described as “commercial caution” in cost analyses. If the revision rates “DR_TTR” are compared with the data of the comparison hospital, the following picture emerges.

The model values:

- Revision rate after one year: 3.32%.
- Revision rate after five years: 8.64%.
- Revision rate after ten years: 15.57%.

The real data values:

- Revision rate after one year: 3.00%.
- Revision rate after five years: 7.90%
- Revision rate after ten years: 13.80%.

The model’s data were again slightly more conservative than the real data. Furthermore, to evaluate the quality of the model, the error probabilities have to be considered. Looking at EP_LTS_G1 it becomes clear that 0.12% of the patients would need a fourth implant. EP_TTR_G1 indicates that 0.43% of patients would require a fourth revision. These probabilities of error are considered to be very low, and therefore the impact on cost would be low as well since the events are in the distant future.

**Fig. 4:**
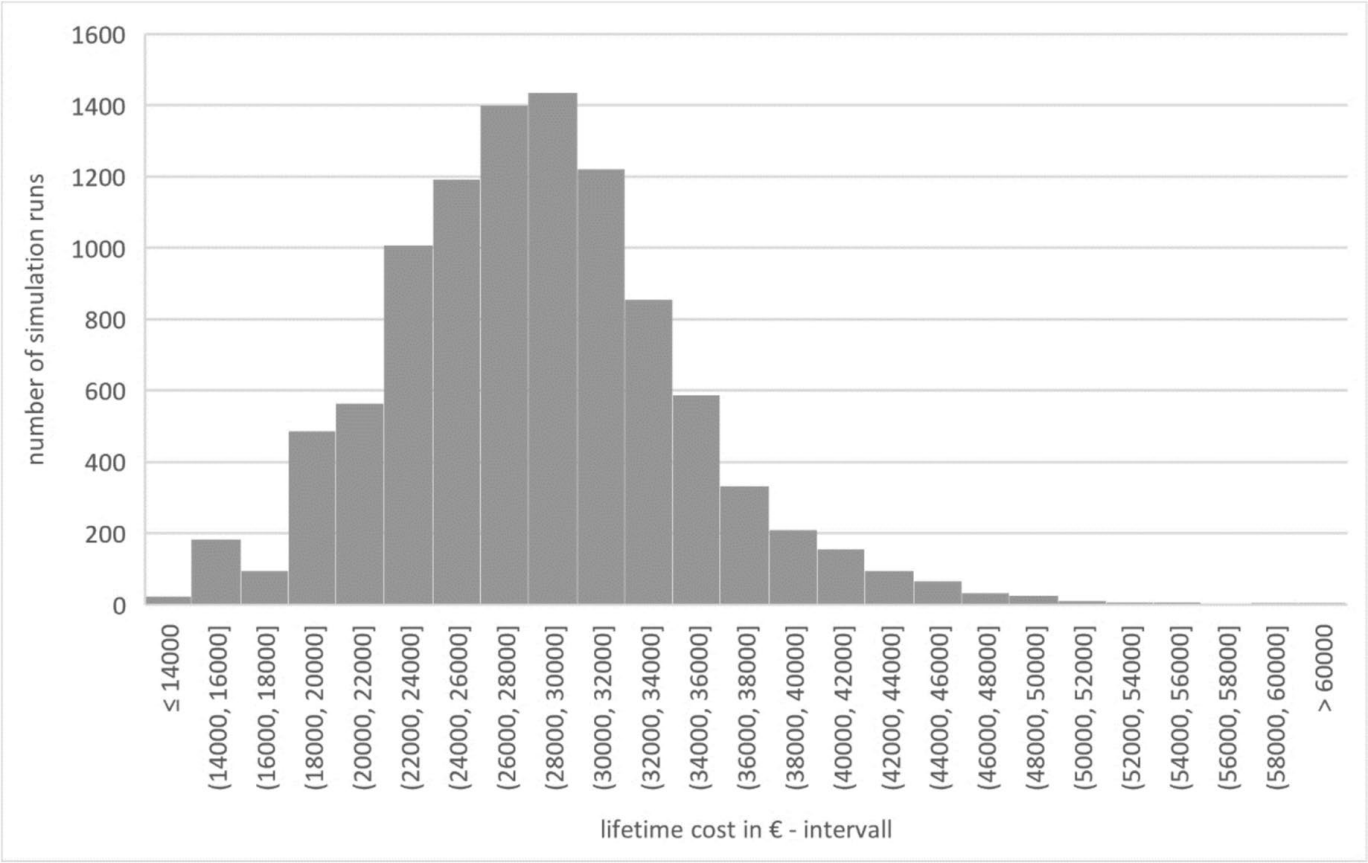
Lifetime cost – group 1 (source: own simulation)

In terms of costs, the lifetime cost (G1_LTC) is 28,324.51€ (± 6120.84€), and the median is 28,076.08€. This is made up of: 14,857.04€ (± 1076.18€) for initial implantation (G1_CFI), 1166.05€ (± 3442.79€) for re-implantations (G1_CRI), 1005.48€ (± 1771.46€) for revisions (G1_CRE), 9399.40€ for processor updates (G1_APU), and 1896.54€ (± 569.58€) for annual follow-up appointments (G1_TSC). Figure 4 shows lifetime costs.

### Model results group 2

**Fig. 5:**
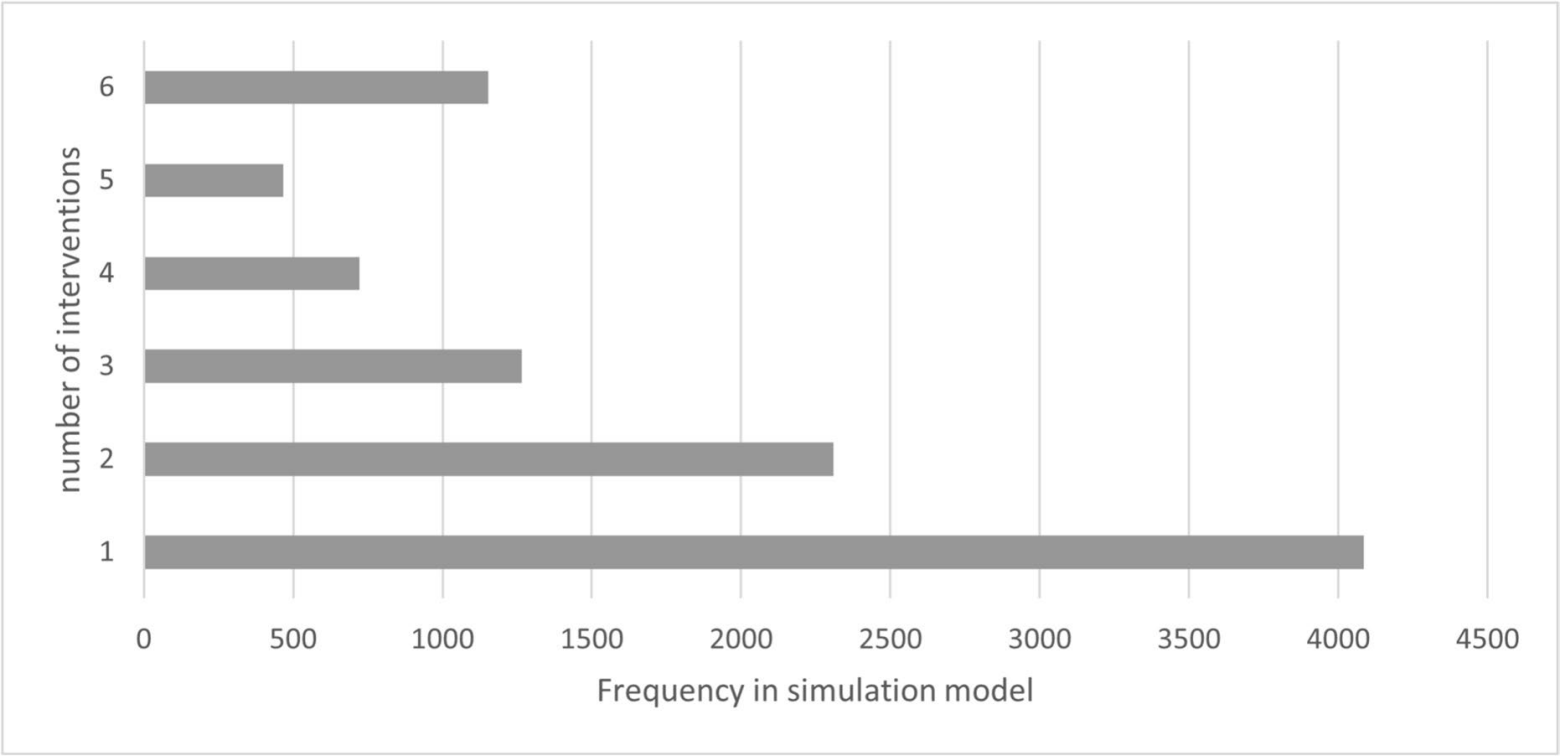
Number of hearing-improving surgeries before VSB implantation – group 2 (source: own simulation)

In group 2, hearing-improvement surgeries preceded VSB implantation. During the care period with hearing-improvement surgeries of 4.01 years (± 5.18), a mean of 2.46 surgeries (± 1.70) including initial intervention were performed. Figure 5 shows the number of surgeries before VSB implantation (maximum limit of six surgeries including initial intervention). It results in EP_NHI_G2 of 8.30%. This means that in 8.30% of cases, more than six interventions would be necessary. However, the model is limited to six inventions to reduce the influence of cost outliers. Furthermore, in the real data the maximum number was also six interventions. Figure 5 shows the number of interventions before VSB implantation.

The further care with VSB implantation values were equivalent to those of group 1. Due to the reduced duration of care, the values for the model errors were slightly reduced. Furthermore, the values for re-implantation and revision requirements decreased. EP_LTS_G2 is 0.10% (previously 0.12%), EP_TTR_G2 was 0.36% (previously 0.43%), NOI_G2 was 1.13 (previously 1.15), NOR_G2 is 0.38 (previously 0.45) and NOP_G2 was 3.06 (previously 3.65).

**Fig. 6:**
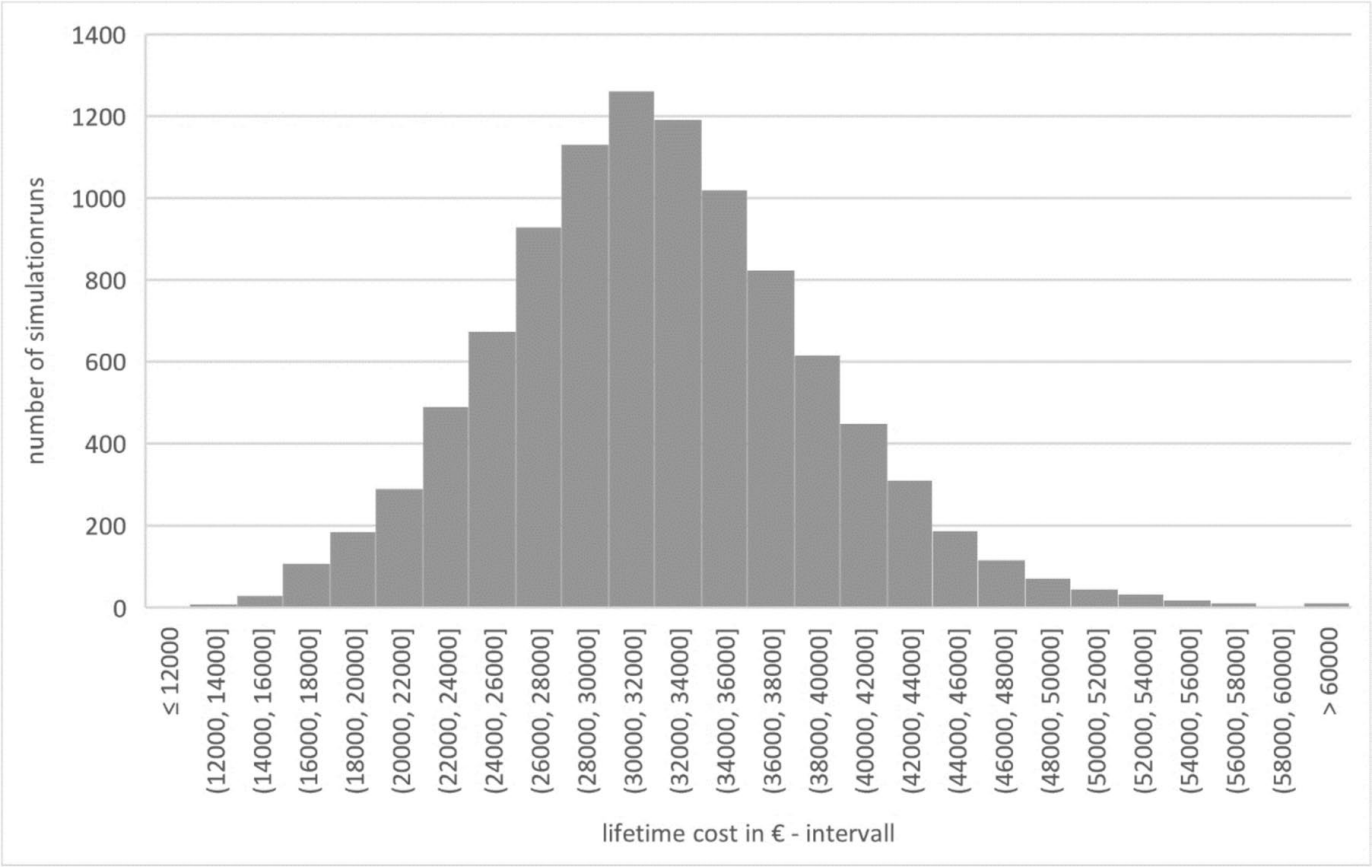
Lifetime cost – group 2 (source: own simulation)

Group 2 lifetime costs (G2_LTC) were 32,187.07€ (± 6901.79€), with a median of 31,849.58€. This comprised 4203.30€ (± 1023.21€) for the initial intervention (hearing-improving surgery) (G2_CFI), 4880.87€ (± 5734.94€) for all subsequent hearing-improving surgeries (G2_CFS), 12,421.78€ (± 2601.63€) for VSB implantation (G2_VSB), 903.21€ (± 2923.00€) for re-implantations (G2_CRI), 774.28€ (± 1503.21€) for revisions (G2_CRE), 7107.08€ (± 4025.18) for processor updates (G2_APU) and 1896.54€ (± 569.58€) for annual check-up appointments (G2_TSC). Figure 6 shows the lifetime costs.

### Model results group 3

Group 3 care consisted exclusively of hearing-improvement surgeries. Assuming a maximum of 15 interventions including initial intervention and a mean time between interventions (DSH) of 2.50 years (± 3.29), the mean number of hearing-improvement surgeries was 10.30 (± 4.26). Problematically, this results in a model error of 25.84%. This means that even with 15 operations, more than 25% of the patients could not be successfully treated over the entire duration (TOP). This results in a care gap of 3.32 (± 7.83) years in the model. Despite this gap in care, the costs for the entire period should be presented.

**Fig. 7:**
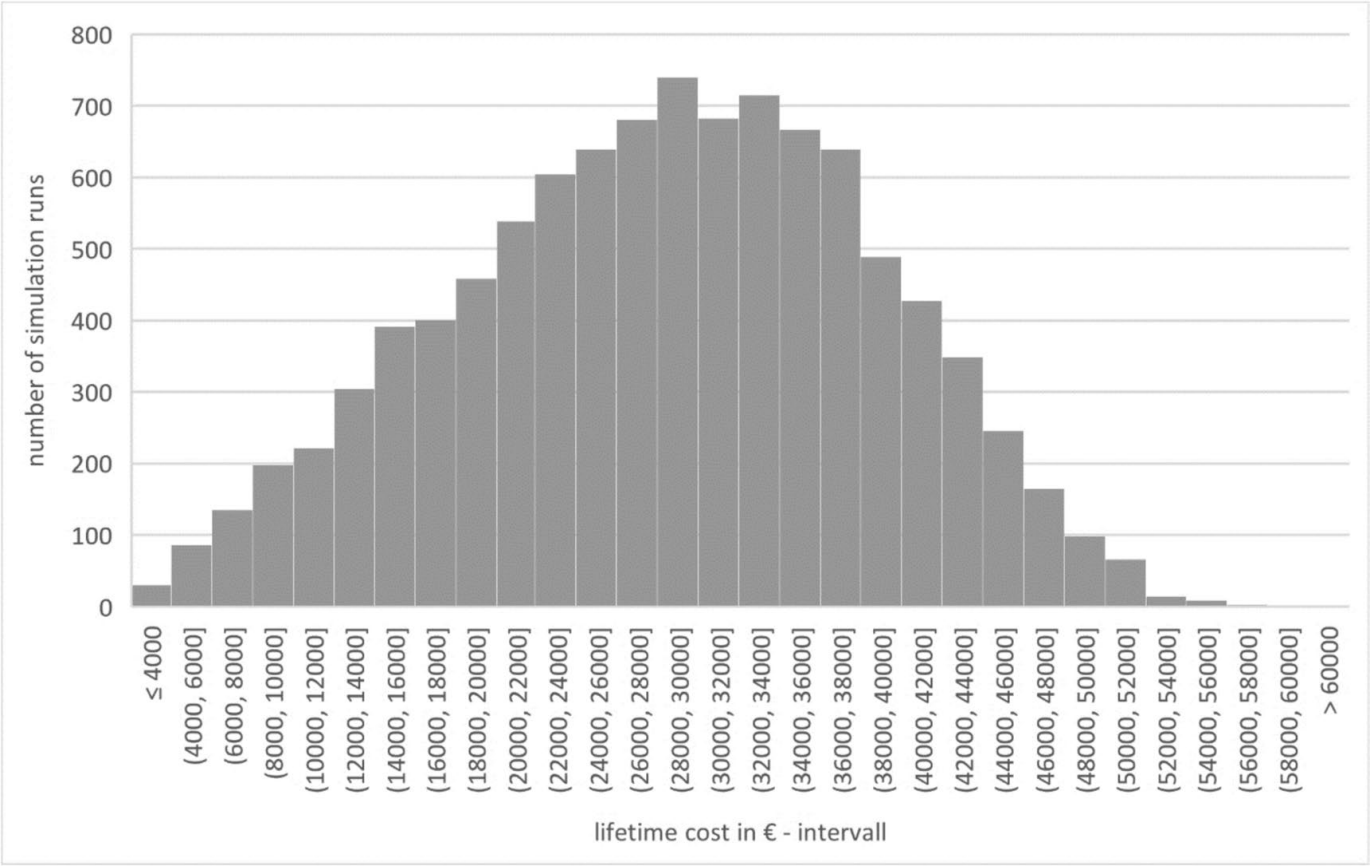
Lifetime cost – group 3 (source: own simulation)

The model shows lifetime costs of 28,381.01€ (± 10,326.73€), with a median of 28,846.55€. Of this, 4203.30€ (± 1023.21€) is for the initial intervention (hearing-improvement surgery) (G3_CFI), 22,281.25€ (± 9962.74€) for all subsequent hearing-improvement surgeries (G3_CFS) and 1896.54€ (± 569.58€) for the annual follow-up appointments (G3_TSC). Figure 7 shows lifetime costs.

The overall model comparison results in the following lifetime costs for the three defined groups:

- Group 1: VSB direct 28,324.51€ (± 6120.84€).
- Group 2: hearing-improvement surgery, then VSB 32,187.07€ (± 6901.79€)
- Group 3: only hearing-improvement surgery 28,381.09€ (± 10,326.73€)

Group 1 generates the lowest costs on average. However, it must be taken into account that there are significant parameters influencing the costs. This is made clear by the standard deviation. The discussion will focus on the relevant influencing parameters. However, as mentioned in the results, not all patients (especially in group three) can be treated to the end of their lives, and the following list represents the costs per successfully treated year. This is the sum of all costs of all simulation runs divided by the sum of all simulated life years with successful treatment.

- Rank 1: VSB direct 1059.61€ per year
- Rank 2: Only hearing-improvement surgery 1212.55€ per year
- Rask 3: Hearing-improvement surgery, then VSB 1229.14€ per year

## Discussion

When evaluating lifetime costs in relation to duration of care, it is important to consider the homogeneity of these costs. The number of hearing-improvement surgeries a patient underwent prior to VSB implantation should be kept in mind. Group 3 in this study showed us that in addition to examining the average costs incurred over an average observation period, further differentiated analysis is necessary. The 25.84% gap presented already has a very high maximum number of interventions (15), which points to the fact that both the duration of care as well as the number of hearing-improvement surgeries are decisive factors in evaluating the individual care alternatives.

### Influence of the duration of treatment (TOP)

**Fig. 8:**
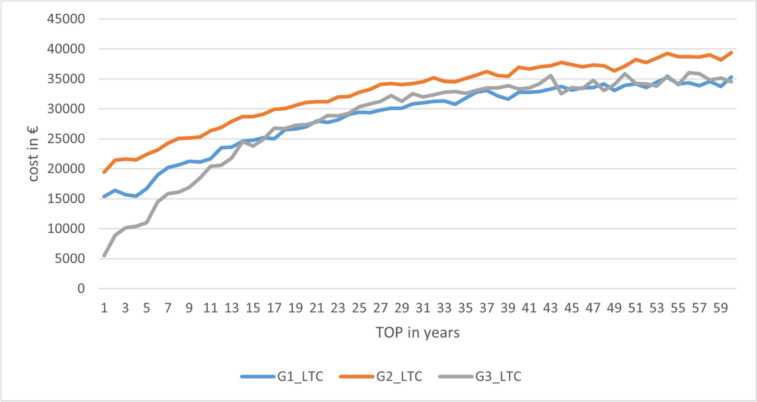
Lifetime cost depending on the duration of treatment (TOP) (source: own simulation)

The advantageousness of the options clearly depends on the duration of care. The mean costs of group 3 (G3_LTC) and group 1 (G1_LTC) intersect between year 16 and 17. Figure 8 shows the cost trend.

Subsequently, the cost curves of groups 3 and 1 are relatively close, with the mean value mostly in favour of group 1. However, the model errors also need to be considered. Thus, the cost trend for group 3 also includes patients for whom the provision of hearing-improvement surgery was not possible until the end of life (25.84%). This circumstance occurs mostly with long remaining lifetimes. This also explains the cost trends with high “TOP” values. Consequently, focusing on the dimension “costs per successful year of care as a function of the total duration of care” appears to be much more target-oriented.

**Fig. 9:**
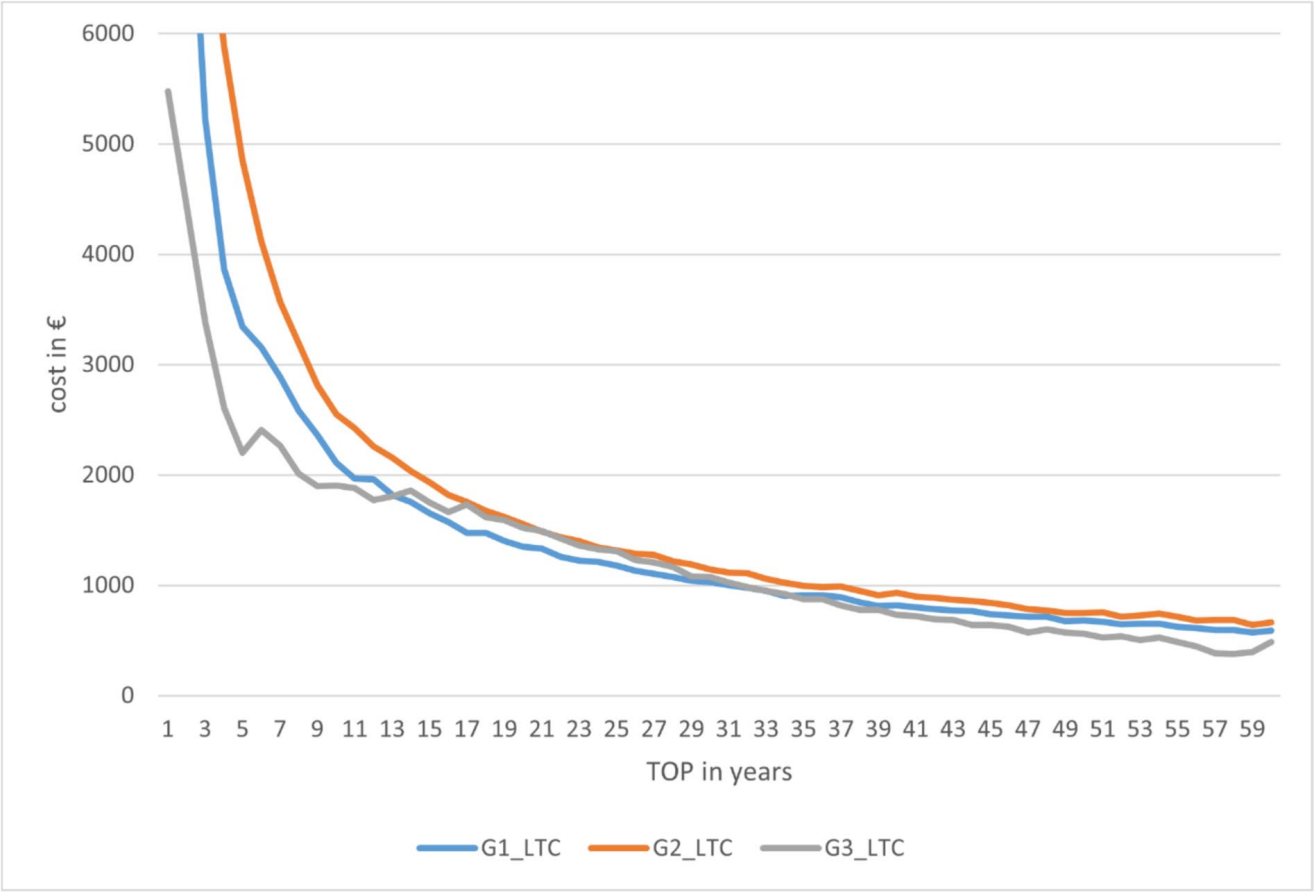
Cost per successful year of care depending on the total duration of treatment (TOP) (source: own simulation)

If only the successfully supplied years are considered, the point of advantage shifts to the 14th year. This shows that there are patients in the model who can be successfully treated in group 3 for less than 14 years. Thus, the option of direct VSB implantation already appears advantageous with a remaining lifetime of 14 years on average. Figure 9 shows the data.

**Fig. 10:**
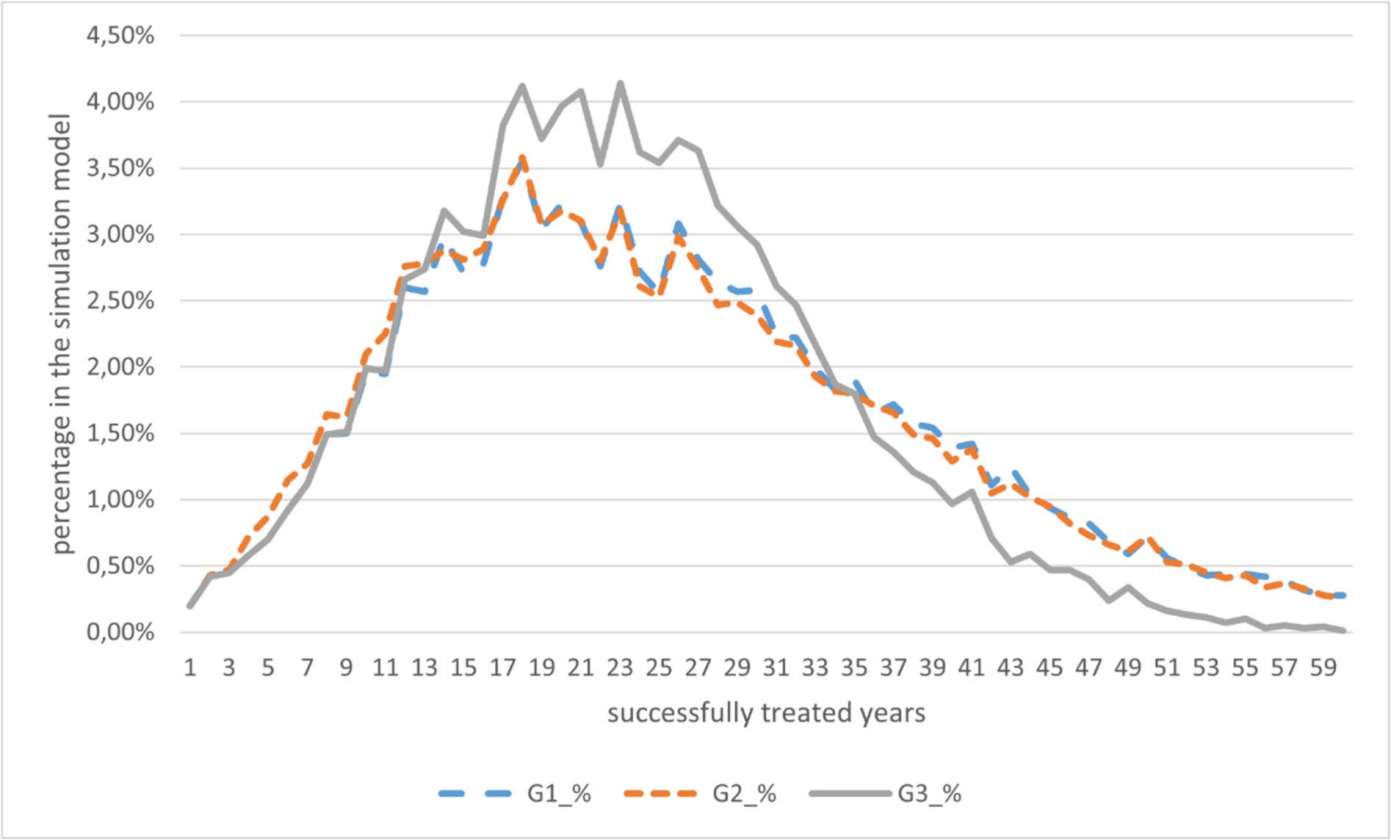
Distribution of successfully treated years in the simulation model. (source: own simulation)

Looking at the data from the 35th year onward, at first glance the costs in group 3 appear to be the lowest. However, this only includes people who can be provided with hearing-improvement operations over a long period of time. Therefore, only the results of patients in which the operations were successful for a relatively long period of time were included. It is important to note how heavily each group is populated in the model (see Fig. 10). While the distribution of G1 reflects the age distribution in the model (TOP), G2 is slightly influenced by the sixth hearing-improvement surgery before VSB implantation, which was not considered. However, the data from G3 show significantly lower durations of successful care. It is clear that the proportion of patients who can be successfully treated decreases from the 35th year onwards.

Figure 10 clearly shows that when the duration of care exceeds 35 years, the likelihood of providing end-of-life care with hearing-improving surgery decreases sharply. This would indicate a switch to a different form of therapy. It therefore seems sensible to choose an alternative treatment for patients with long residual lifetimes that can be successful over their entire lifetime.

Up to this point, the following conclusions can be derived from the model:

- When considering the duration of care and taking into account a successful treatment, VSB implantation seems to be a reasonable to option for remaining lifetimes of more than 13 years.
- For shorter treatment durations, hearing-improvement surgery appears to make sense from an economical perspective.
- If the remaining lifetime is more than 35 years, successful treatment with hearing-improving interventions alone becomes less likely, as even with the maximum number of 15 hearing-improving operations defined in the model, an increasing proportion of people in this group cannot be treated for the remaining time. Thus, from a cost perspective, alternative treatment options must be considered. The VSB seems to be a reasonable choice due to its lower lifetime costs.

### Homogeneity of costs

With respect to cost homogeneity, the results from the entire simulation as well as the costs for remaining lifetimes of 10, 20 and 30 years will be examined as examples. Table 5 shows the mean costs as well as the homogeneities of the costs. A t-test was used to check whether the mean values are different and if significant differences existed in the standard deviation. A p-value of less than 0.05 indicates significant difference.

**Table 5:** Costs and cost homogeneities depending on the duration of treatment. (source: own simulation)

	Total simulation set (TOP)	10 years	20 years	30 years
n in simulation	10,000	196	323	258
Group 1				
Average cost in €	28,325	21,123	27,037	30,842
Standard deviation in €	6121	3249	4258	4652
Cost homogeneity	0.82	0.87	0.86	0.87
Group 2				
Average cost in €	32,187	25,321	31,096	34,232
Standard deviation in €	6902	4496	5662	5975
Cost homogeneity	0.82	0.85	0.85	0.85
Group 3				
Average cost in €	28,381	18,471	27,409	32,552
Standard deviation in €	10327	8018	9690	8037
Cost homogeneity	0.73	0.70	0.74	0.80
t-test–mean cost				
G1 vs. G2	** < 0.001**	** < 0.001**	** < 0.001**	** < 0.001**
G1 vs. G3	0.637	** < 0.001**	0.528	**0.003**
G2 vs. G3	** < 0.001**	** < 0.001**	** < 0.001**	**0.007**
Bonnet-test - standard deviation				
G1 vs. G2	** < 0.001**	**0.013**	**0.002**	**0.031**
G1 vs. G3	** < 0.001**	** < 0.001**	** < 0.001**	** < 0.001**
G2 vs. G3	** < 0.001**	** < 0.001**	** < 0.001**	** < 0.001**

**Fig. 11:**
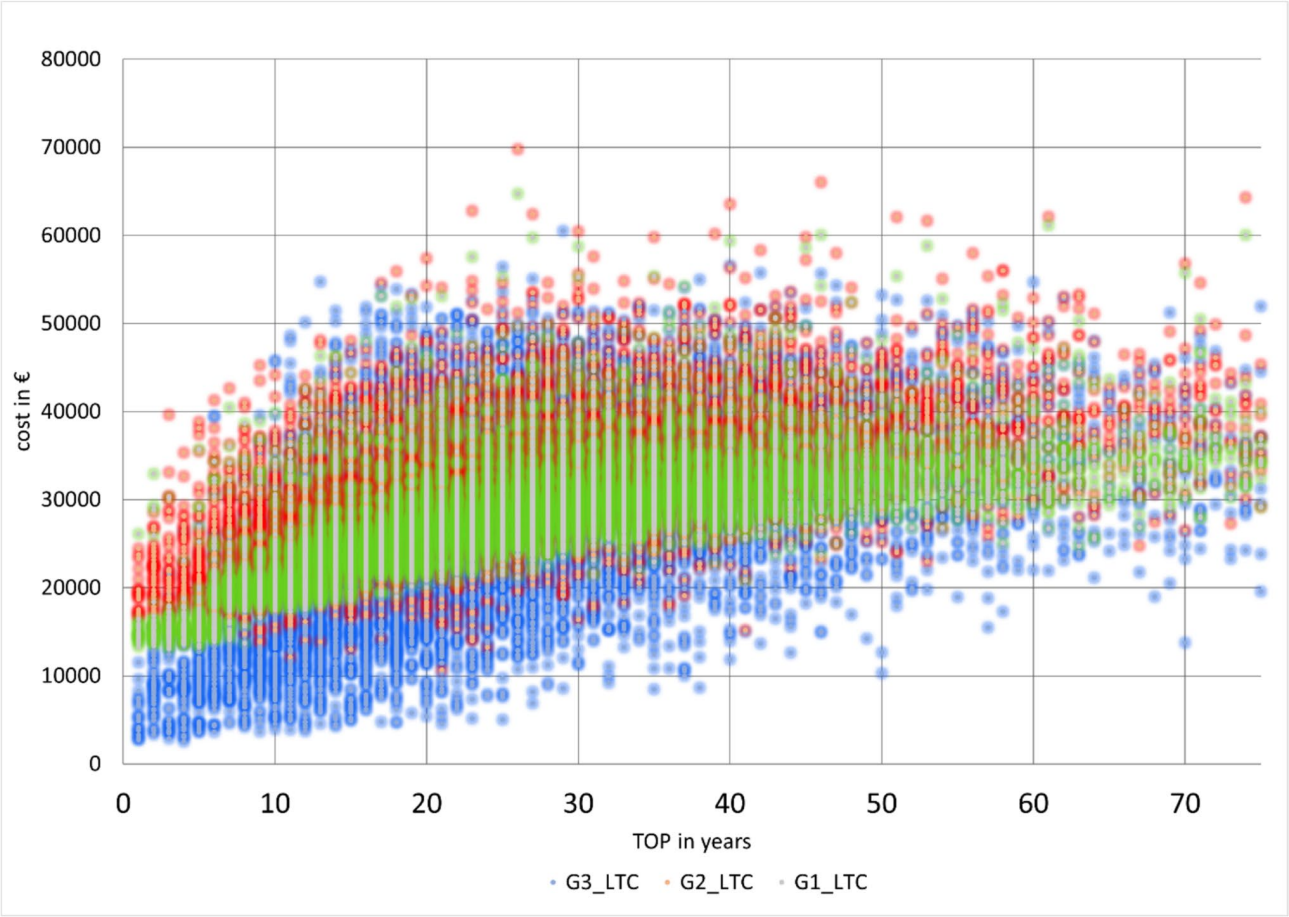
Overview of simulation results (here: G1 green; G2 red; G3 blue) (source: own simulation)

The costs of group 1 and 3 did not differ throughout the entire simulation. This is due to the fact that for short treatment durations G1 dominates, but for long durations group 3 is preferable, as was previously stated. Nevertheless, the differences in the standard deviations were always significant - and thus significantly higher in groups 2 and 3 due to the influence of the hearing-improvement operations. Consequently, treatment with the VSB had significantly higher homogeneity coefficients, attributable to very low mid- and long-term costs, due to small numbers of revision surgeries and re-implantations over the entire period of care. Therefore, the use of the middle ear implant therefore reduces the risk of cost outliers, even though the implantation of an active middle ear implant is often associated with increased complexity and higher costs [27]. The advantage of the lower standard deviation is evident in Fig. 11. Costs in group 3 scatter significantly more than in the other groups.

Furthermore, it should be noted that the analysis presented here excludes the costs of hearing aid fitting, which is often necessary after hearing-improvement surgeries. Hence, lifetime costs of hearing aid fitting, which can sum up to 4518€, as determined by Thum et al. should have been taken into account either in order to comprehensively report total lifetime costs of group 3 [28]. Also, additional treatment costs for the more than 25% of patients in group 3 who couldn’t be successfully treated (e.g. due to ear infections) until the end of life, would have been needed to be considered, too. These 25% of patients could potentially be described as a subset of patients with insufficient benefit from this combination therapy, as even state-of the-art power hearing aids would not be able to provide sufficient gain and/or output required by these patients [29]. For these 25% of patients who couldn’t be successfully treated in group 3, bypassing the middle ear and air–bone gap (ABG) with a powerful active middle ear implant or a direct acoustic stimulation of the cochlea, might have been the more promising hearing solution [29].

From an economic point of view, the following aspect can be added:

– The use of a middle ear implant makes future costs significantly easier to plan, since there is a higher degree of cost homogeneity.

### Influence of the number of hearing-improvement surgeries

Another question to be addressed regards the influence of the number of hearing-improvement surgeries administered. We aimed to find out whether the average costs depend on the number of hearing-improvement operations. Table 6 shows the mean costs and standard deviations. A t-test was used to determine significance. A p value of less than 0.05 indicates that the costs were significantly increased compared to “one surgery less”.

**Table 6:** Influence of the number of hearing-improvement operations on lifetime costs (source: own simulation)

NHI (G1 or G2 data)	Mean in €	Standard deviation	Cost increased?
“0” = G1_LTC	28,325	6121	**–**
“1”	30,606	6499	** < 0.001**
“2”	32,347	6493	** < 0.001**
“3”	32,796	6953	**0.030**
“4”	34,067	7567	** < 0.001**
“5”	35,394	7361	** < 0.001**

The costs increase significantly as the number increases. In the simulation, the difference between two and three hearing-improvement operations is the smallest. This is due to the age distribution in the simulation. Here it becomes clear that with a relatively low remaining life expectancy, an additional third surgery can postpone VSB implantation so long that no processor upgrade will be necessary shortly before death. However, it has already been shown in the previous discussion points that group 3 seems to be advantageous at low life expectancies.

From an economic perspective, the following can be stated: switching to VSB implantation seems to be reasonable even after hearing-improvement surgeries have already been performed, because each subsequent hearing-improvement surgery significantly increases the lifetime costs.

**Fig. 12:**
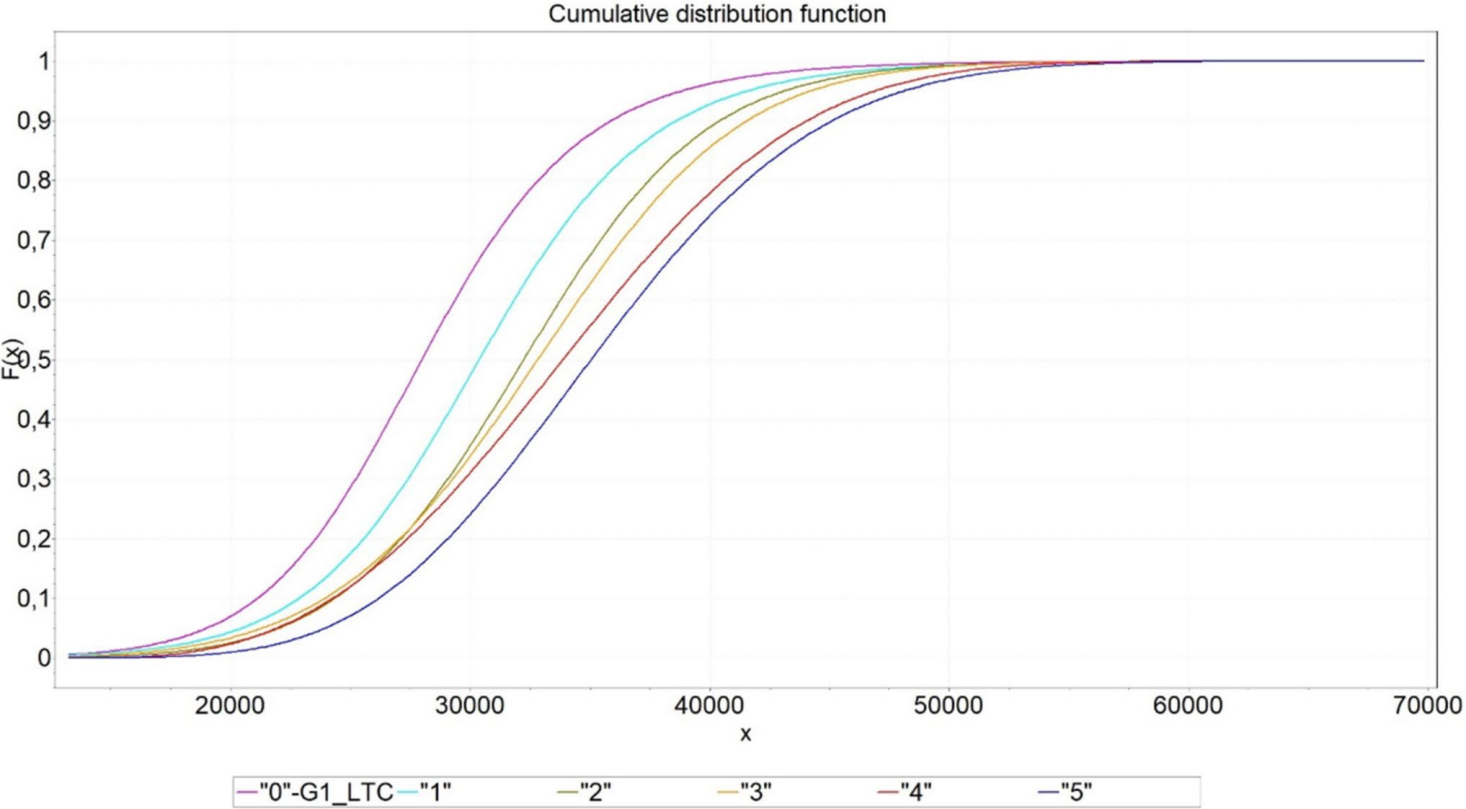
Cumulative distribution functions of the costs for the treatment alternatives “0 to 5 hearing-improvement operations prior to VSB implantation” (source: own simulation)

Figure 12 shows the distribution functions, which is helpful for discussion of more than just the mean values. These distribution functions were determined by distribution identification from the simulation results.

“0” – G1_LTC – Gen. Logistic k=0.06296 s=3351.4 m=27,976.0

“1” – Gen. Logistic k=0.04093 s=3582.5 m=30,364.0

“2” – Burr k=2.0806 a=7.2139 b=36,652.0

“3” – Burr k=3.1797 a=6.2471 b=41,144.0

“4” – Weibull (3P) a=2.9827 b=23,244.0 g=13,307.0

“5” – Johnson SB g=2.3776 d=3.3915 l=1.1399E+5 x=−2760.1

In about 80% of cases, fewer hearing-improvement operations incur less costs. Patients with five hearing-improvement operations always result in the highest costs. In approximately 25% of cases, the difference between two, three or four hearing-improvement operations is unclear. This effect again mainly results in patients with a low remaining life expectancy, where a change to the VSB is not cost-effective. However, one hearing-improvement operation always appears to be better than two or more. This option is dominated only by the direct VSB option.

From an economic point of view, it can be concluded that if the remaining life expectancy is sufficient, a switch to VSB treatment after the first hearing-improvement surgery seems to be reasonable.

Based on the previous aspects of the discussion, it can be concluded:

1. When the life expectancy is less than 14 years, the most favorable is a hearing-improving surgery.
2. In patients with residual lifetimes of more than 35 years, the success of exclusive treatment with hearing-improvement surgery is unlikely. Since it is shown that a low number of hearing-improvement operations results in lower lifetime costs, direct VSB implantation appears to be a good choice.
3. If the remaining lifespan is between 14 and 36 years, a definitive statement about the advantageousness is difficult, but results show that downscaling hearing-improving operations makes sense.

The following is an example of a possible economic recommendation.

1. If TOP < 14 (age at first intervention > 67), then G3 (only hearing-improvement surgery)
2. If TOP > 35 (age at first intervention < 46), then direct G1 (VSB implantation)
3. If 13 < TOP < 36, then hearing-improvement surgery (age at first intervention 46 to 67). Change to VSB after one surgery if remaining lifetime at date of new intervention > 13 years (corresponds to maximum age of 67) (= G2 with hearing-improving surgery), otherwise remain at G3 (only hearing-improvement surgery).

Based on the age distribution of TOP, we have four different patient groups. 16.46% of patients can be exclusively treated with hearing-improvement surgeries due to their age at first intervention (> 67). 5.93% of patients also receive only hearing-improvement surgeries due to their age at second invention being 68 or older. 23.76% of patients can undergo direct VSB implantation because their age at first intervention is less than 46 years. 53.85% of patients can first receive hearing-improvement surgery, followed by a VSB.

Such a strategy leads to a mean cost of 29,099.50€ with a standard deviation of 8384.29€. This is still significantly above the cost of direct VSB implantation, but below the cost of hearing-improvement surgery followed by VSB implantation (each p < 0.001).

The main effect results in the number of years the patient is successfully treated. The costs per successfully treated year of life are 1088.61€. This is still higher than the cost of VSB (1059.61€) per year, but the model also only considers cases in which a hearing-improvement surgery is followed by a second intervention. However, the costs are reduced by more than 10% compared to the other strategies.

## Conclusion

Choosing a treatment option is a complex medical decision. Treatment success and outcome for the patient is difficult to assess from the “economists’ desk”. Cost data, probabilities from medical data sets and knowledge of treatment processes enable us to estimate future costs. This can support medical decision-making, which is also often influenced by short-term cost aspects.

Our study has shown that time plays a decisive role. We found that differentiated decision making can reduce lifetime costs, and treatment alternatives perceived as costly today may be beneficial in the long run. Direct, first-line VSB implantation or implantation after a low number of hearing-improvement surgeries is a reasonable treatment option. This opens up the possibility of a stronger patient-centered approach in decision-making. When focusing on the current costs of a single intervention, often only the option of a (further) hearing-improving surgery remained open due to the refusal of coverage by health insurance. However, if the total lifetime costs are considered, a broader set of treatment alternatives (direct implantation or first hearing-improving surgery) may result for the patient (taking into account his or her age), which can also be considered economically advantageous.

From an economic point of view – in particular from the point of view of health insurance companies – our analysis shows that the determination of lifetime costs is an important basis for economic decision-making that goes beyond the individual financial year of the health insurance company. Based on patient data, it was clearly shown that it is economically feasible to consider the option of VSB implantation earlier.

With regard to the methodology of our analysis, it should be noted that cost analyses are always subject to the limitation that the input parameters can change or that different health care systems may have different cost structures. Furthermore, data on events in the distant future, whether medical or economic, is often subject to uncertainties. Future events are therefore difficult to calculate. However, the uncertainty can be reduced by using simulation models that take distribution functions into account. It should also be noted that a conservative approach in the inclusion of unclear events such as complications reduces uncertainty.

The focus of this analysis was on the cost of treatment. Although “successfully treated years” were addressed based on the times between surgeries, the quality of care in these years was not considered. Other methods of health economic evaluation relate treatment outcomes to costs. Conceivable outcomes include factors such as decibels (dB) or gains in quality of life (e.g., QALYs). What these analyses have in common is that knowledge about costs is necessary. Thus, the present results can be regarded as a starting point for further analyses, methodologically as well as for the current object of investigation, VSB implantation.

## Data Availability

The data that support the findings of this study are available from the corresponding author, upon reasonable request.
